# Blue Light Supplemented at Intervals in Long-Day Conditions Intervenes in Photoperiodic Flowering, Photosynthesis, and Antioxidant Properties in Chrysanthemums

**DOI:** 10.3390/antiox11122310

**Published:** 2022-11-22

**Authors:** Jingli Yang, Jinnan Song, Byoung Ryong Jeong

**Affiliations:** 1 Department of Horticulture, Division of Applied Life Science (BK21 Four), Graduate School of Gyeongsang National University, Jinju 52828, Republic of Korea; 2 Institute of Agriculture and Life Science, Gyeongsang National University, Jinju 52828, Republic of Korea; 3 Research Institute of Life Science, Gyeongsang National University, Jinju 52828, Republic of Korea

**Keywords:** antioxidant ability, photoperiodism, photosynthetic carbon assimilation, supplemental blue light, unusual photoperiod

## Abstract

The flowering of chrysanthemum (*Chrysanthemum morifolium* Ramat.), inhibited by long-day lighting, can be reversed with a short period of low supplemental blue light (S-BL). Both flowering and the reactive oxygen species (ROS) scavenging processes are primarily driven by sugars created by photosynthetic carbon assimilation. In addition, the antioxidant ability potentially affects flowering in photoperiod- and/or circadian rhythm-dependent manners. This indicates that there is an interactive relationship among blue (B) light, photosynthetic efficiency, sugar accumulation, and antioxidant ability in flowering regulation. Here, 4 h of 30 μmol·m^−2^·s^−1^ photosynthetic photon flux density (PPFD) S-BL was applied at the end of a 13-h long-day period (LD13 + 4B) at different intervals during 60 days of experimental duration. The five experimental groups were named according to the actual number of days of S-BL and their intervals: applied once every day, “60 days-(LD13 + 4B) (100.0%)”; once every other day, “30 days-(LD13 + 4B) (50.0%)”; once every three days, “15 days-(LD13 + 4B) (25.0%)”; once every five days, “10 days-(LD13 + 4B) (16.7%)”; and once every seven days, “7 days-(LD13 + 4B) (11.7%)”. Two non-S-BL control groups were also included: 60 10-h short days (60 days-SD10) and 13-h long days (60 days-LD13). At the harvest stage, varying degrees of flowering were observed except in “60 days-LD13” and “7 days-(LD13 + 4B) (11.7%)”. The number of flowers increased and the flower buds appeared earlier as the proportion of S-BL days increased in LD13 conditions, although the “60 days-SD10” gave the earliest flowering. The proportion of initial, pivotal, and optimal flowering was 16.7% (“10 days-(LD13 + 4B)”), 50.0% (“30 days-(LD13 + 4B)”), and 100.0% (“60 days-(LD13 + 4B)”), respectively. Meanwhile, a series of physiological parameters such as the production of enzymatic or non-enzymatic antioxidants, chlorophyll content, photosynthetic efficiency, enzyme activities, and carbohydrate accumulation were significantly improved by “30 days-(LD13 + 4B) (50.0%)” as a turning point until the peaks appeared in “60 days-(LD13 + 4B) (100.0%)”, as well as the expression of florigenic or anti-florigenic and some antioxidant-synthetic genes. Furthermore, the results of principal component analysis (PCA) indicated that S-BL days positively regulated flowering, photosynthesis, carbohydrate accumulation, and antioxidant production. In aggregate, the pivotal and optimal proportions of S-BL days to reconcile the relationship among flowering, photosynthetic carbon assimilation, and antioxidant ability were 50.0% and 100.0%, respectively. However, there are still significant gaps to be filled in order to determine the specific involvement of blue light and antioxidant abilities in flowering regulation.

## 1. Introduction

The commercially and ornamentally important plant, chrysanthemum (*Chrysanthemum morifolium* Ramat.) is a qualitative short-day plant (SDP), where a sufficiently long night over a certain critical level is absolutely required for flowering. The flowering characteristic that depends on day-length variation is “photoperiodism” [1]. Thus, the seasonal variations in the day length and light have become a major obstacle to their flowering. To fit the anti-seasonal demands in horticultural markets, the cultivation of seasonal plants in a plant factory with artificial lighting (PFAL) become more popular, providing controlled lighting conditions and removing hostile environmental variables [2]. Light-emitting diodes (LEDs) are professionally supplied to plant factories for growth lighting and are more power-economical and customizable than conventional lighting systems. LED treatments with specific irradiation wavelengths are expanding in floriculture and horticulture [3].

Plants identify the light quality with around three groups of photoreceptors: phytochromes, cryptochromes, and at least one unidentified ultraviolet light receptor(s). The absorbance peaks of phytochromes are in red (R, 600 to 700 nm) and far red (FR, 700 to 800 nm) light, and to a lesser extent in blue (B, 400 to 500 nm) light. Blue light is also absorbed by cryptochromes. Cryptochromes, especially those found in Brassicaceae, have a role in the suppression of stem elongation [4]. Blue light has been shown to decrease extension growth, particularly in hypocotyls and epicotyls [5,6,7]. Blue light contributes to flower induction in several long-day plants (LDPs), including *Hyoscyamus niger* L. [8] and *Arabidopsis thaliana* Heynh. [9]. Moreover, researchers have experimented with using different wavelengths of light to stimulate the flowering of SDPs in a long photoperiod. SDPs, as previously said, are unable to flower when the day is too long. However, short-day plants, chrysanthemum and kalanchoe, flowered in long-day conditions when supplied with low levels of monochromatic blue light [10,11,12,13,14]. In these situations, blue light supplementation did not impair the blossom initiation. However, relatively little is known about how blue light regulates flowering.

The light quality affects the photosynthesis, and therefore the sugar accumulation. In addition to providing energy to plants, sugars also function as signaling molecules within them, thus regulating growth and development [15]. Capsicum [16], lettuce [17], and strawberry [18] were found to contain the highest levels of sugars when treated with mixed R and B LEDs. However, with LED treatments, sugar concentration was reduced in Chinese cabbage [19] and oak-leaf lettuce [20]. There are a wide variety of metabolic activities within plants that continuously produce reactive oxygen species (ROS), including superoxide radicals and hydrogen peroxide [21]. Ascorbate peroxidase, glutathione reductase, superoxide dismutase, catalase, and ascorbate dismutase are enzymatic antioxidants that regulate the ROS levels [22,23]. Non-enzymatic antioxidants such as carotenoids, tocopherols, flavonoids, ascorbic acid, and phenolic compounds also regulate the ROS levels [24]. In plants treated with certain LEDs, photooxidative stress has been found to increase antioxidant levels, including many phytonutrients. Under high-PPFD B, R, and FR mixed LEDs, red pak choi and tatsoi seedlings had greater amounts of ascorbic acid and α-tocopherol [25]. Lettuce [26] and Gynura [27] showed increased antioxidant activity when exposed to LED treatments with increasing quantities of blue light. Increased flavonoid and phenolic contents were also seen in rose, chrysanthemum, and campanula cultivated with mixed R and B LED treatments with greater blue light proportions [28]. Tomato seedlings grown under B LEDs also presented the highest antioxidant activities [24,29]. The levels of phenolics and flavonoids in tomato seedlings grown under B and white (W) LEDs were generally excellent [29]. Pea seedlings grown under R LEDs, on the other hand, showed the greatest antioxidant activity [30], demonstrating a cultivar-dependent response to LED light.

Flowering is primarily driven by sugars created from photosynthetic carbon assimilation [13]. Plant flowering is also affected by light quality-related photosynthetic efficiency and carbon absorption, which is related to altered carbon and nitrogen metabolism [31,32]. Thus, light quality can be used to regulate flowering by affecting photosynthesis. Furthermore, plants evolved sophisticated and well-coordinated molecular and metabolic networks to counteract abiotic stressors, with sugar metabolism playing an important role [33]. Sugar buildup has a role in both ROS production and scavenging processes [34]. Sugars and ROS can have an indirect association via nicotinamide adenine dinucleotide phosphate (NADPH)-producing metabolisms, such as the oxidative pentose-phosphate pathway, or a direct relationship with sugars serving as genuine ROS scavengers [35]. Hence, sugar-involved flowering and sugar-related antioxidant abilities are potentially controlled by light-hosted photosynthetic efficiency and carbon assimilation. Moreover, one study found that the non-enzymatic antioxidant ascorbic acid functions as a co-factor in the biosynthesis of gibberellic acid (GA) and abscisic acid (ABA), influencing not only the endogenous level but also the signaling of these plant hormones, and thus affecting the developmental flowering and senescence in a photoperiod- and/or circadian rhythm-dependent manner [36]. However, how ascorbic acid or other antioxidants control flowering requires further investigation.

In the current research, an innovation of supplemental LED blue light (S-BL) was provided for the horticulture and floriculture industry. The S-BL is innovatively applied at different intervals in a long-day period to understand the efficient proportions of S-BL days to the whole experimental duration in chrysanthemum flowering regulation. According to our results, (1) putting forward more possible options of proportions of S-BL days in initial, pivotal, and optimal flowering, respectively, (2) providing effective and optimal proportions of S-BL days to reconcile the relationship among flowering, sugar accumulation, and antioxidant properties, (3) and supporting flexibility of blue light application based on actual demands, will reduce the cultivation cost, save energy, and comply with the purpose of sustainable development. Our study could inspire researchers and growers to choose the appropriate LED lighting options for flowering control, phytochemical contents, nutritional values, etc.

## 2. Materials and Methods

### 2.1. Plant Materials and Growth Conditions

The pot experiment was carried out at Gyeongsang National University in Jinju, Korea, in a closed-type plant factory (770.0 cm long, 250.0 cm wide, and 269.5 cm high, Green Industry Co. Ltd., Changwon, Republic of Korea). In this work, the ornamental species chrysanthemum (*Chrysanthemum morifolium* Ramat.) “Gaya Glory”, a qualitative SDP, was used as the experimental material. Flowers Breeding Research Institute, Gyeongnam Agricultural Research & Extension Services, Changwon, Gyeongnam, Korea, provided rooted cuttings with 8 ± 2 leaves per plant, which were separately transplanted into 10 cm plastic pots with a commercial medium (BVB Medium, Bas Van Buuren Substrates, EN-12580, De Lier, The Netherlands), one plant per pot. After planting, plants were moved to this closed–type plant factory and acclimated to 23/18 °C (light/dark), 70 ± 10% RH, and 270 ± 5 μmol·m^−2^·s^−1^ PPFD supplied with F48T12-CW-VHO fluorescent lamps (Philips Co., Ltd., Eindhoven, The Netherlands). A pressurized gas tank supplied CO_2_ to complement plant photosynthesis on time, and an electrolyte CO_2_ sensor (Model No. GMT220 Carbocap, Vaisala, Vantaa, Greater Helsinki, Uusimaa, Finland) monitored online maintained a concentration of 350 ± 50 parts per million (PPM). The air circulation system in this location was designed with the fans uniformly spaced so that the conditioned air could blow horizontally into the developing rooms through several regularly spaced openings. The plants were treated with photoperiodic light treatments after one week of acclimation (with 16-h LD). Daily irrigation with a multipurpose nutrient solution (macro-elements: Ca^2+^, Mg^2+^, K^+^, NH_4_^+^, NO_3_^−^, SO_4_^2−^, and H_2_PO_4_^−^; microelements: B, Cu, Fe, Mn, Mo, and Zn; pH = 6.5) [14] was carried out from 8:30~9:30 a.m. Additionally, a three-replication randomized complete block design with ten plants in each replication, with a total of 30 plants in each treatment randomly arranged among replications was used to reduce the effects of light location in an opaque compartment.

### 2.2. Photoperiodic Light Treatments

**Figure 1:**
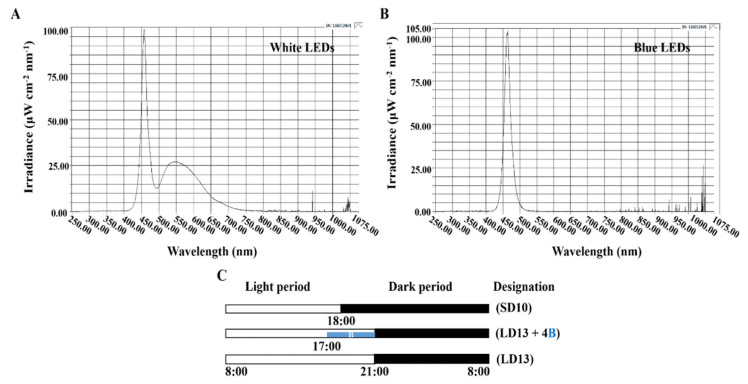
The spectral distribution of the experimental light treatments (**A**,**B**): the daily white light (~400–720 nm and peaked at 435 nm) provided by white LEDs and blue light (peaked at 450 nm) from blue LEDs used as the supplemental light. The experimental light schemes employed in this study (**C**): the light period started every day at 8:00 a.m.; plants in the control groups were treated with a 10-h short-day or 13-h long-day condition, without any blue light; the 4-h blue light (4B) with 30 μmol·m^−2^·s^−1^ PPFD was used to supplement the white light at the end of the LD13 (LD13 + 4B).

Based on previous studies [12,13], 10-h short-day (SD10) and 13-h long-day (LD13) treatments without any supplemental blue light (S-BL) were established as the two control groups in the current study. The light duration was started every day at 8:00 a.m. Plants were grown with daily light at an intensity of 300 ± 5 μmol·m^−2^·s^−1^ PPFD provided by white (W) MEF50120 LEDs (More Electronics Co. Ltd., Changwon, Korea) with a wide spectrum ranging from 400 to 720 nm and a distinct peak at 435 nm in blue (Figure 1A). Because the photoperiodic flowering and physiology of chrysanthemums are sensitive to the 30 μmol·m^−2^·s^−1^ PPFD of S-BL [13], the B LEDs (450 nm) (Figure 1B) were used to provide 4-h supplemental blue light (30 μmol·m^−2^·s^−1^ PPFD) at the end of LD13 (LD13 + 4B) (Figure 1C) at different intervals. The detailed description and abbreviation of the light treatments and the proportion of S-BL days in the 60 days of experimental duration are shown in Table 1. Light distribution was recorded at 1 nm wavelength intervals using a spectroradiometer (USB 2000 Fiber Optic Spectrometer, Ocean Optics Inc., Dunedin, FL, USA; detects wavelength between 200 to 1000 nm) and the uniformity was verified by measuring the light intensity at three points of each light treatment at the canopy level through a quantum radiation probe (FLA 623 PS, ALMEMO, Holzkirchen, Bavaria, Germany).

### 2.3. Measurements of Growth Parameters

The plant growth parameters, such as plant height, number of branches, leaves, and flowers per plant, leaf area per leaf, and plant dry weight, were collected after 60 days of photoperiodic light treatments. The days to visible flower buds in each treatment were determined by counting the number of days from initiating the light treatment to the date when the first flower bud appeared. The number of flowers per plant contained both blooming flowers and visible flower buds at the harvest stage. The leaves with a length > 1 cm were counted to determine the total number of leaves per plant. The top fourth true leaves were selected to measure the fully expanded leaf area using a leaf area meter (Li-Cor 3000, Li-Cor, Lincoln, NE, USA). After careful cleaning, divided samples of shoots were oven-dried (the drying oven, Venticell-222, MMM Medcenter Einrichtungen GmbH., Munich, Germany) at 85 °C for 5~7 days until a constant mass was reached to determine the dry weight. Additionally, the harvested samples were immediately placed in liquid nitrogen and then stocked in a −80 °C refrigerator for the subsequent physiological investigations.

### 2.4. Chlorophyll Content

The leaf chlorophyll (Chl) content was determined according to Lichtenthaler and Buschmann [37]. After 60 days of photoperiodic light treatments and at 9:00 a.m., 0.2 g of fresh leaf samples were collected from the top fourth mature leaves in the main stem counting from the apex and ground, and liquid nitrogen was used to extract the chlorophyll in 2 mL 80% (*v*/*v*) acetone overnight at 4 °C until the leaf samples were completely discolored. A UV spectrophotometer (Libra S22, Biochrom Ltd., Cambridge, UK) was used for colorimetry at A_663 nm_ and A_646 nm_. The pigment contents were calculated from the following equations: chlorophyll a (Chl a) = 12.25 × A_663_ − 2.79 × A_646_; chlorophyll b (Chl b) = 21.50 × A_646_ − 5.10 × A_663_. For each experiment, 3 technical and 6 biological replicates were performed.

### 2.5. Measurements of Photosynthesis and Chlorophyll Fluorescence

A leaf porometer (SC-1, Decagon Device Inc., Pullman, WA, USA) was used to measure the net photosynthetic rate (*P*n), transpiration rate (*T*r), stomatal conductance (*G*s), and intercellular CO_2_ concentration (*C*i) of the top fourth completely expanded adult leaf for each plant at harvest time. The average of six measurements taken on each leaf was utilized. To maintain the same stable circumstances, these parameters were monitored in the closed-type plant factory from 9:00 to 11:00 a.m.

A photosystem (Fluor Pen FP 100, Photon Systems Instruments, PSI, Drásov, Czech Republic) was used to quantify the leaf chlorophyll fluorescence. As previously stated, the top fourth fully expanded adult leaf from each plant was chosen for these measures. After dark-adapting the leaves for 30 min with a leaf clip, a 0.6 s saturating light pulse (3450 μmol·m^−2^·s^−1^ PPFD) was applied to obtain the maximum fluorescence (*F*m) and minimum fluorescence (*F*0). The leaf was then light-adapted for 5 min with continuous actinic light (300 μmol·m^−2^·s^−1^ PPFD, identical to the growth environment) with saturating pulses every 25 s, and the maximum light-adapted fluorescence (*F*m′) and steady-state fluorescence (*F*s) were measured. The maximal PSII quantum yield (*F*v/*F*m) was calculated as *F*v/*F*m = (*F*m − *F*0)/*F*m [38]. To produce minimum fluorescence after PSI excitation (*F*0′), the actinic light was switched off and a far-red pulse was delivered. *F*v′/*F*m′ = (*F*m′ − *F*s)/*F*m′ was used to compute the photochemical efficiency (*F*v′/*F*m′) of PSII. Furthermore, the photochemical quenching coefficient (*qP*) was determined to be *qP* = (*F*m′ − *F*s)/(*F*m′ − *F*0′) [39]. For each experiment of photosynthesis or chlorophyll fluorescence, 3 technical and 6 biological replicates were performed.

### 2.6. Accumulation of Carbohydrates and Soluble Proteins

Weighed measurements of 0.3 g of −80 °C stocked leaf samples were collected at 10:00 p.m. after 60 days of the photoperiodic light treatments and the anthrone colorimetric technique was used to determine the amounts of starch and soluble sugars [40,41]. Stocked leaf samples were collected and promptly submerged in liquid nitrogen before being crushed into a fine powder over an ice bath for the total soluble protein extraction. In 50 mM PBS (1 mM EDTA, 1 mM polyvinylpyrrolidone, and 0.05% (*v*/*v*) triton-X, pH 7.0), 0.1 g of the powder was homogenized. The resultant mixture was then centrifuged (13,000 rpm, 4 °C, 20 min) to extract the supernatant, which was then utilized for the total protein estimation and enzyme activity assays. The total protein assessments were led by [42]. A UV spectrophotometer (Libra S22, Biochrom Ltd., Cambridge, UK) was used to assess the concentrations of soluble sugars, starch, and soluble proteins at A_630 nm_, A_485 nm_, and A_590 nm_, respectively. For each experiment, 3 technical and 6 biological replicates were performed.

### 2.7. Enzyme Activities

The preceding step’s total protein solution was utilized to examine the enzymatic activities, which were quantified using a UV spectrophotometer (Libra S22, Biochrom Ltd., Cambridge, UK). Three technical and six biological replicates were conducted for each enzymatic measurement. At 34 °C for 1 h, sucrose synthase (SS) and sucrose phosphate synthase (SPS) were measured in a 1-mL reaction mixture comprising of a 500 μL enzyme extract. A 300 μL 30% (*v*/*v*) KOH solution was added to this combination, which was then put in a 100 °C water bath for 10 min before being progressively cooled to room temperature. After applying a 200 μL 0.15% (*v*/*v*) anthrone-sulfuric acid solution, the mixture was incubated at 40 °C for 20 min while the enhancement of A_620 nm_ was measured. The phosphoenolpyruvate carboxykinase (PEPC) was assayed in a 1 mL reaction mixture consisting of 50 mM Tris-HCl (pH 8.0), 5 mM MnCl_2_, 2 mM DTT, 10 mM NaHCO_3_, 0.2 mM NADH, 5-unit NAD-MDH, and a 160 μL enzyme extract. To begin the process, 2.5 mM phosphoenolpyruvate was added (PEP). In a 1.5 mL reaction mixture comprising of 100 mM imidazole-HCl (pH 7.5), 50 mM KCl, 10 mM MgCl_2_, 0.05% (*w*/*v*) BSA, 2 mM DTT, 150 μM NADH, 1-unit LDH, 2 mM ADP, and a 150 μL enzyme extract, the phosphoenolpyruvate phosphatase (PEPP) was measured. The reaction was started with 2 mM PEP, and the change in A_412 nm_ was measured. The total activity of Ribulose 1,5-diphosphate carboxylase/oxygenase (RuBisCO) was determined by injecting 100 μL of the supernatant into 400 μL of an assay mixture containing 50 mM Tris-HCl (pH 8.0), 5 mM DTT, 10 mM MgCl_2_, 0.1 mM EDTA, and 20 mM NaH_14_CO_3_ (2.0 GBq mmol^−1^) at 30 °C. After a 5-min activation time, the reaction was started by adding RuBP to 0.5 mmol L^−1^ and was stopped after 30 s with 100 μL of 6 mol L^−1^ HCl. Moreover, the activities of soluble starch synthase (SSS), adenosine diphosphate glucose pyro-phosphorylase (ADPGPPase), and uridine diphosphate glucose pyro-phosphorylase (UDGPPase) were measured according to the protocol described by Doehlert et al. [43] and Liang et al. [44]. The enzymatic activity measurements mentioned above were conducted in accordance with the directions provided by Yang et al. [45] and Feng et al. [46].

The activities of enzymes that scavenge reactive oxygen species (ROS) were also investigated. According to Aebi [47], the catalase (CAT) activity in homogenates was tested by directly measuring the induction of H_2_O_2_ at A_240 nm_. The CAT activity was calculated using the H_2_O_2_ extinction coefficient (40 mM^−1^ cm^−1^). Castillo et al. [48] presented a technique for assessing the activity of guaiacol peroxidase (GPX). The absorbance at A_470 nm_ was calculated using an extinction value of 26.6 mM^−1^ cm^−1^. Becana et al. [49] investigated the ability of superoxide dismutase (SOD) to inhibit the photochemical reduction of nitroblue tetrazolium (NBT). One unit of SOD was defined as the amount of enzyme that provided 50% inhibition of NBT decrease by monitoring at A_560 nm_. According to Nakano and Asada [50], the activity of ascorbate peroxidase (APX) was measured for 1 min at A_290 nm_ (extinction coefficient 2.9 mM^−1^ cm^−1^). Three technical and six biological duplicates were conducted for each measurement.

### 2.8. Extraction and Colorimetric Assays of Non-Enzymatic Antioxidants

To obtain carotenoids, 1 g of frozen leaf samples was milled with 1 g of quartz and 15 mL of acetone containing 1% BHT (2,6-di-tert-butyl-4-methylphenol) [51]. The mixture was shaken for 30 min at 4 °C in the dark before being centrifuged for 10 min at 17,000 rpm. The supernatant was discarded, and the material was re-extracted until it became colorless. The supernatants were pooled. The total carotenoid content was determined using a spectrophotometric technique published by Rodriguez-Amaya [52], which involved measuring the absorbance at 450 nm using a UV spectrophotometer (Libra S22, Biochrom Ltd., Cambridge, UK). The results were expressed as “mg” of carotenoids per gram of frozen weight.

In search of anthocyanins, 1 g of frozen leaf samples was crushed along with 1 g of quartz and 15 mL of 1% HCl in methanol [53]. After shaking for 2 h at room temperature, the mixture was incubated overnight in the dark at 20 °C before centrifugation at 4000 rpm for 15 min. The supernatant was collected, and the samples were extracted three times in methanol with 15 mL of 1% HCl. The supernatants were pooled and diluted as needed for the analyses. The pH differential technique [54] was used to determine the total anthocyanin levels. The extract was diluted in pH 1.0 and pH 4.5 solutions (0.1 M HCl and 25 mM KCl) (0.4 M CH_3_COONa). Using a UV spectrophotometer, the absorbance of the mixtures was measured at 535 and 700 nm in comparison to pure water (Libra S22, Biochrom Ltd., Cambridge, UK). The value (Abs_535_ − Abs_700_)_pH1.0_ − (Abs_535_ − Abs_700_)_pH4.5_ corresponds to the absorbance due to the anthocyanin. Results were expressed as “mg” of kuromanin equivalents (KuE) per gram of frozen weight.

For the compound ascorbic acid, 4 g of frozen leaves were crushed along with 1 g of quartz and 80 mL of the extraction solution (20 g/L HPO_3_). The mixture was mixed for 1 h at 4 °C before being centrifuged for 15 min at 15,000 rpm. The concentration of reduced ascorbic acid was determined using the association of vitamin chemical 2,6-dichloroindophenol (DCIP) technique [55]. In brief, each molecule of vitamin C changed a molecule of DCIP into a molecule of DCIPH2, and the conversion was tracked using a UV spectrophotometer as a drop in the absorbance at 520 nm (Libra S22, Biochrom Ltd., Cambridge, UK). The results were expressed as “mg” of ascorbic acid per gram of frozen weight.

For total flavonoids, 1 g of frozen leaf samples was crushed with 1 g of quartz and 10 mL of 100% acetone. The mixture was mixed for 1 h at 70 °C before being centrifuged for 15 min at 17,000 rpm. The supernatant was discarded, and the material was extracted again using the same process but only incubating for 15 min. The supernatants were pooled and diluted as needed for the analyses. Perva-Uzunali et al. [56] provided the exact extraction procedure. Colorimetric analysis was used to determine the flavonoid content [57]. A combination of 200 μL extract and 150 μL sodium nitrite (NaNO_2_) (5% *w*/*v*) was first incubated at room temperature for 6 min. After that, 150 μL of AlCl_3_·6H_2_O (10% *w*/*v*) was added and incubated at room temperature for 6 min. 800 μL of NaOH (10% *w*/*v*) solution was added and incubated at room temperature for 15 min. The extract was replaced with distilled water for the blank. A UV spectrophotometer (Libra S22, Biochrom Ltd., Cambridge, UK) was used to measure the absorbance at 510 nm. The total flavonoid was expressed in “mg” Quercetin equivalent (QE) per gram of frozen weight.

To determine the total phenolics, the procedures for aliquoting the leaf extract based on well-established references [58,59,60] were used. The phenolic content was evaluated with a colorimetric analysis [61,62]. 200 μL of leaf aliquot extract, 800 μL deionized water, and 100 μL of Folin–Ciocalteu reagent were mixed and incubated for 3 min at room temperature. 300 μL of Na_2_CO_3_ (20% *w*/*v*) was added and incubated for 2 h at room temperature in the dark. A UV spectrophotometer (Libra S22, Biochrom Ltd., Cambridge, UK) was used to measure the absorbance at 765 nm. Instead of aliquot extract, distilled water was used to make a blank. The total phenolics were expressed in “mg” of gallic acid equivalent (GAE) per gram of frozen weight.

### 2.9. Real-Time Quantitative PCR Verification

The total RNA was extracted using the RNeasy Plant Mini Kit (Takara Bio Inc., Tokyo, Japan) and processed with RNase-free DNase (Takara Bio Inc., Tokyo, Japan) as directed by the manufacturer. A PrimeScript^®^ Reverse Transcriptase (Takara Bio Inc., Tokyo, Japan) was used to synthesize cDNA from 1 μg of total RNA, in accordance with the manufacturer’s instructions. The cDNA was diluted 10-fold, and 5 μL of it was utilized in a 15-μL quantitative RT-PCR (qRT-PCR) experiment using SYBR Premix Ex Taq™ II (Takara Bio Inc., Tokyo, Japan), which was carried out on a Roche Light Cycler 96 real-time fluorescence quantitative PCR apparatus (Roche, Basel, Switzerland). The relative expression levels of each gene were determined using the 2^−∆∆Ct^ method [63]. In our investigation, the chrysanthemum homologues of *Arabidopsis* were denoted as “*Cm* + *gene*” The data were averaged and standardized against the expression of the reference genes *CmACTIN* and *CmEF1* (elongation factor 1α). The primer sequences and PCR conditions used in the analyses are listed in Table 2. For each experiment, 3 technical and 6 biological replicates were performed.

### 2.10. Principal Component Analysis

For the flowering-, photosynthesis-, carbohydrate-, or antioxidant-related parameters, the principal component analysis (PCA) was conducted on the three replicates, using the MINITAB 16.2.1 statistical software, aiming to extract trends when multiple qualitative variables were used, formulating new variables correlated to the original ones [71]. The PCA outputs included treatment component scores as well as variable loadings [72].

### 2.11. Statistical Analysis

All plants in our study were picked at random. Excel 2016 and the DPS software package (DPS for Windows, 2009) were used to process, plot, and statistically analyze the data. Significant differences among the treatments were assessed by an analysis of variance (ANOVA), followed by the Duncan’s multiple range test at a probability (*p*) ≤ 0.05 with a statistical program (SAS, Statistical Analysis System, V. 9.1, Cary, NC, USA). The differences between each treatment were tested by Student’s *t*-test (*p*) ≤ 0.05. Moreover, the experimental assays used to obtain all results were repeated six times and presented as the mean ± standard error.

## 3. Results

### 3.1. Flowering and Growth Parameters

**Figure 2:**
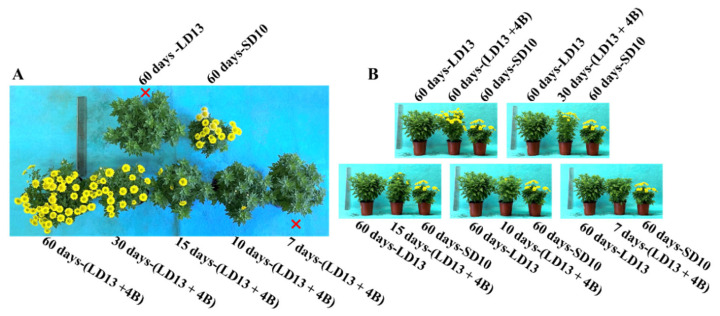
The morphology of chrysanthemum “Gaya Glory” under different treatments at the harvest stage. Photographs from the top (**A**) and side (**B**) view, respectively. The red cross (❌) demarcates non-flowered plants. See Figure 1 and Table 1 for details of the experimental treatments.

In the current study, the 4-h supplemental blue light (S-BL) supplied at different intervals in the LD13 period significantly affected the photoperiodic flowering, morphology, and growth of “Gaya Glory” plants (Figure 2 and Table 3). Firstly, the flowering of chrysanthemums strongly responded to the unusual photoperiodic light treatments. At the harvest stage, other than the treatments of “60 days-LD13” and “7 days-(LD13 + 4B)”, flowering to varying degrees was observed (Figure 2A). Notably, the unusual photoperiodic blue light treatments intervened in the flowering of these SDPs which would otherwise have not flowered in the long-day conditions. As the number of days of S-BL application increased, flower buds appeared earlier. Among all experimental groups, flowering occurred significantly earlier in the treatment “60 days-(LD13 + 4B)” although the “60 days-SD10” (one of the control groups) gave the earliest flowering (Table 3). Similarly, the number of flowers increased as the days of S-BL increased. A pivotal turn in the flower number began in the “30 days-(LD13 + 4B)” treatment, and the “60 days-(LD13 + 4B)” produced the greatest number of flowers. Except for the non-flowered treatments (“7 days-(LD13 + 4B)” and “60 days-LD13”), the changing pattern of the flower number per plant was “60 days-(LD13 + 4B)” > “30 days-(LD13 + 4B)” > “60 days-SD10” > “15 days-(LD13 + 4B)” = “10 days-(LD13 + 4B)”. Overall, the flowering in chrysanthemums responded differently to different proportions of the number of days of S-BL during the whole experimental duration (60 days). The proportions in initial, pivotal, and optimal flowering were 16.7% (“10 days-(LD13 + 4B)”), 50.0% (“30 days-(LD13 + 4B)”), and 100.0% (“60 days-(LD13 + 4B)”), respectively.

Secondly, we also investigated how the growth and morphology of chrysanthemums responded to the different number of days of S-BL in the photoperiodic light treatments. As shown in Figure 2B and Table 3, the plants grown under LD13 conditions are normally taller than those grown under SD10 environments, and the shoot height roughly increased as the proportion of days of S-BL increased. However, there were no significant differences in the shoot length among treatments of “60 days-(LD13 + 4B)”, “30 days-(LD13 + 4B)”, “15 days-(LD13 + 4B)”, and “60 days-LD13”. Moreover, our results showed that the blue light helped improve the plant’s dry weight and leaf area, especially in the LD13 conditions. As the proportion of S-BL days increased, the dry weight and leaf area also gradually increased, and the “60 days-(LD13 + 4B)” treatment produced the greatest dry weight and leaf area. (Table 3). Additionally, LD13 conditions with S-BL appeared to be more favorable in inducing leaves and branches, and the non-flowered treatments of “60 days-LD13” and “7 days-(LD13 + 4B)” resulted in the greatest number of branches and leaves among all treatments (Table 3). The changing patterns of the number of leaves and branches can be summarized as follows. The number of flowers seems to be inversely proportional to the number of branches or leaves, indicating a kind of competitive relationship between the flower induction and leaf or branch formation.

### 3.2. Chlorophyll Content

**Figure 3:**
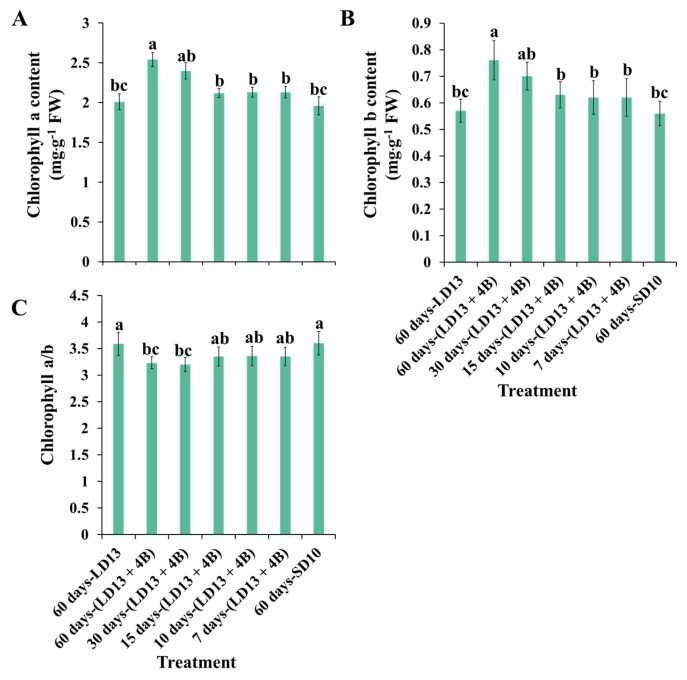
The content of chlorophyll a (**A**), chlorophyll b (**B**), and the ratio of chlorophyll a to b (**C**) of “Gaya Glory” grown with different intervals of supplemental blue light, after 60 days of exposure to the photoperiodic light treatments. Vertical bars indicate the means ± standard error (*n* = 6). Different lowercase letters indicate significant separation within treatments by the Duncan’s multiple range test at *p* ≤ 0.05. See Figure 1 and Table 1 for details of the experimental treatments.

The chlorophyll (Chl) content of chrysanthemum leaves was considerably affected by the different proportions of S-BL days (Figure 3). In this experiment, increasing the number of days of S-BL from 0 to 60 increased the contents of Chl a and b, while the Chl a/b decreased, which may be due to the substantial production of Chl b. Moreover, the chlorophyll a and b contents were always the highest in “60 days-(LD13 + 4B)”, which was slightly higher than those in “30 days-(LD13 + 4B)”. In addition, the chlorophyll content of chrysanthemum leaves grown in “15, 10, and 7 days-(LD13 + 4B)” was found to be insignificantly different, but always higher than those in “60 days-LD13 and SD10” treatments. These improvements suggest that reaching the optimal proportion of days of S-BL could effectively stimulate Chl production and accumulation in plants. In our case, such a proportion of S-BL days is at least 50.0% (“30 days-(LD13 + 4B)”), which can effectively promote Chl synthesis and accumulation.

### 3.3. Photosynthetic Index and Chlorophyll Fluorescence

Table 4 shows the photosynthetic and chlorophyll fluorescence characteristics of chrysanthemum plants in response to the different proportions of S-BL days. The *P*n value increased as the number of days of S-BL increased. The maximum *P*n value of chrysanthemum leaves appeared in “60 days-(LD13 + 4B)”. This increase in the net photosynthetic rate indicates that the number of days of blue light application is positively related to the leaf development and chlorophyll content in chrysanthemum plants, and further improves the photosynthetic efficiency. “60 days-(LD13 + 4B)” induced the greatest values of *T*r, *G*s, and *C*i, and the lowest values were observed in the two control treatments “60 days-LD13 and SD10”.

A similar trend of *F*v’/*F*m’ or *qP* was observed: “60 days-(LD13 + 4B)” > “30 days-(LD13 + 4B)” > “15 days-(LD13 + 4B)” = “10 days-(LD13 + 4B)” > “7 days-(LD13 + 4B)” = “60 days-LD13” > “60 days-SD10”. However, there were insignificant differences in the *F*v/*F*m among all treatments due to the un-stressful environments, were only “60 days-(LD13 + 4B)” and “30 days-(LD13 + 4B)” showed slightly increased *F*v/*F*m values (Table 4). Overall, 50.0% (“30 days-(LD13 + 4B)”) or a higher proportion of S-BL days significantly improved the light energy conversion efficiency of the PSII reaction center and enhanced the actual light energy capture efficiency. Eventually, the treatments of “60 days-(LD13 + 4B)” and “30 days-(LD13 + 4B)” led to a greater *P*n among all treatments.

### 3.4. Contents of Carbohydrates and Soluble Proteins

**Figure 4:**
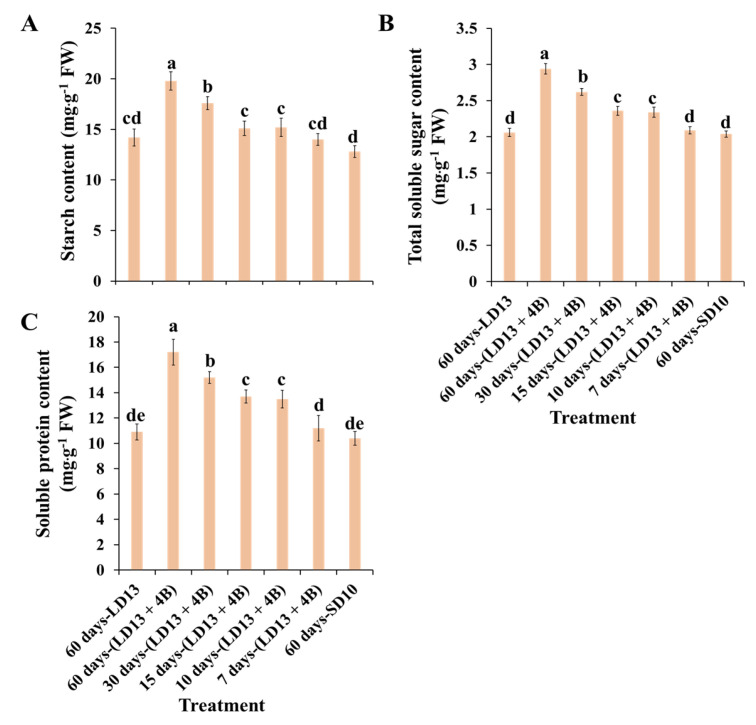
The contents of starch (**A**), total soluble sugars (**B**), and total soluble proteins (**C**) of “Gaya Glory” under different intervals of supplemental blue light, after 60 days of exposure to the photoperiodic light treatments. Vertical bars indicate the means ± standard error (*n* = 6). Different lowercase letters indicate significant separation within treatments by the Duncan’s multiple range test at *p* ≤ 0.05. See Figure 1 and Table 1 for details of the experimental treatments.

The accumulation of carbohydrates and soluble proteins differently responded to various proportions of S-BL days in photoperiodic treatments (Figure 4). According to quality evaluation, treatment “60 days-(LD13 + 4B)” caused the greatest organic nutrient accumulation in chrysanthemum plants which tightly correlated with the abundant chlorophyll content and highly activated PSII. Moreover, photosynthetic organic matter production and its accumulation, as well as mineral transport, provide indispensable nutrients for flower bud production and differentiation, which is consistent with the finding that “60 days-(LD13 + 4B)” induced the greatest number of flowers. Interestingly, the proportion of S-BL days reaching 50.0% (“30 days-(LD13 + 4B)”) appears to be a pivot, where proportions greater than this greatly improve the growth and development of plants, as observed with the accumulation of carbohydrates and soluble proteins in our study.

### 3.5. Enzymatic Activities of Carbohydrate Synthesis- or Photosynthesis-Related Enzymes

**Figure 5:**
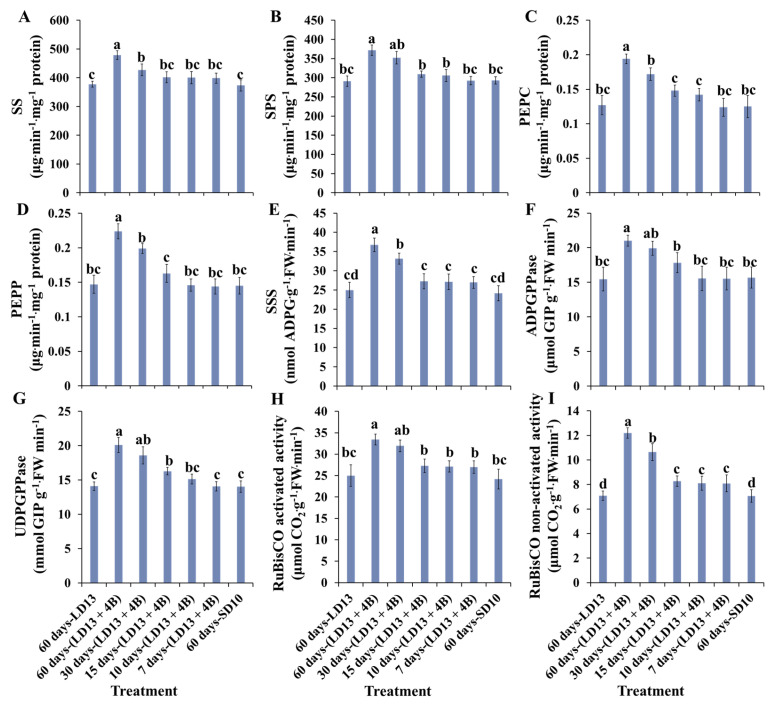
The carbohydrate synthesis- or photosynthesis-related enzymatic activities of “Gaya Glory” under different intervals of supplemental blue light, after 60 days of exposure to the photoperiodic light treatments. Sucrose synthesis enzymes: (**A**) sucrose synthase (SS), (**B**) sucrose phosphate synthase (SPS), (**C**) phosphoenolpyruvate carboxykinase (PEPC), and (**D**) phosphoenolpyruvate phosphatase (PEPP). Starch synthesis enzymes: (**E**) soluble starch synthase (SSS), (**F**) adenosine diphosphate glucose pyro-phosphorylase (ADPGPPase), and (**G**) uridine diphosphate glucose pyro-phosphorylase (UDGPPase), and photosynthesis-related enzyme: the activated (**H**) and non-activated (**I**) activities of Ribulose 1,5-diphosphate carboxylase/oxygenase (RuBisCO). Vertical bars indicate the means ± standard error (*n* = 6). Different lowercase letters indicate significant separation within treatments by the Duncan’s multiple range test at *p* ≤ 0.05. See Figure 1 and Table 1 for details of the experimental treatments.

The activities of carbohydrate synthesis- or photosynthesis-related enzymes in chrysanthemums to explore the response to various proportions of S-BL days in photoperiodic treatments were further investigated (Figure 5). In general, the sucrose synthesis-related enzymes (sucrose synthase (SS), sucrose phosphate synthase (SPS), phosphoenolpyruvate carboxykinase (PEPC), and phosphoenolpyruvate phosphatase (PEPP)) and starch synthesis-related enzymes (soluble starch synthase (SSS), adenosine diphosphate glucose pyro-phosphorylase (ADPGPPase), and uridine diphosphate glucose pyro-phosphorylase (UDGPPase)) responded to the application of S-BL. However, the “60 days-(LD13 + 4B)” was the best-performing treatment. A similar response of total activities (both activated and non-activated) of Ribulose 1,5-diphosphate carboxylase/oxygenase (RuBisCO) to S-BL was observed as above. This facilitation was significantly reduced when the proportion of S-BL days was lower than 50.0% (“30 days-(LD13 + 4B)”).

### 3.6. Antioxidant Ability

**Figure 6:**
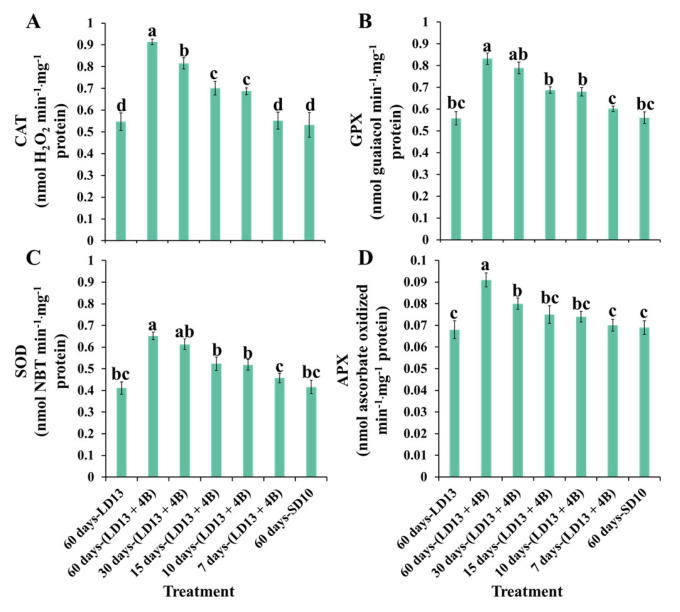
The enzymatic antioxidants of “Gaya Glory” under different intervals of supplemental blue light, after 60 days of exposure to the photoperiodic light treatments. The reactive oxygen species (ROS) scavenging enzymatic activities: (**A**) catalase (CAT), (**B**) guaiacol peroxidase (GPX), (**C**) superoxide peroxidase (SOD), and (**D**) ascorbate peroxidase (APX). Vertical bars indicate the means ± standard error (*n* = 6). Different lowercase letters indicate significant separation within treatments by the Duncan’s multiple range test at *p* ≤ 0.05. See Figure 1 and Table 1 for details of the experimental treatments.

The proportion of S-BL days in the total photoperiodic light treatment also significantly influenced the antioxidant ability. Figure 6 presents the enzymatic antioxidants of “Gaya Glory” under different intervals of S-BL. Generally, the activities of reactive oxygen species (ROS)-scavenging enzymes (CAT, GPX, SOD, and APX) in chrysanthemum leaves positively responded to the proportion of S-BL days. Still, the “60 days-(LD13 + 4B)” was the best-performing treatment. From our results, the S-BL was an effective promoter of expediting the redundant ROS processing inside plant cells, and this facilitation in enzymatic antioxidants was significantly increased when the proportion of S-BL days was higher than 50.0% (“30 days-(LD13 + 4B)”).

**Figure 7:**
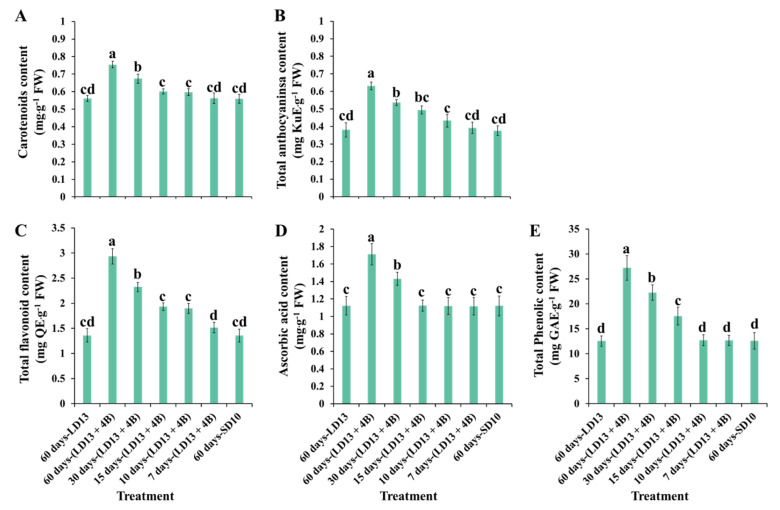
The non-enzymatic antioxidants of “Gaya Glory” under different intervals of supplemental blue light, after 60 days of exposure to the photoperiodic light treatments. Vertical bars indicate the means ± standard error (*n* = 6). Different lowercase letters indicate significant separation within treatments by the Duncan’s multiple range test at *p* ≤ 0.05. See Figure 1 and Table 1 for details of the experimental treatments.

Figure 7 shows the investigation of non-enzymatic antioxidants of “Gaya Glory” under different intervals of S-BL, after 60 days of exposure to the unusual photoperiodic light treatments. As expected, the facilitation in non-enzymatic antioxidant production was significantly increased when the proportion of S-BL days was over 50.0% (“30 days-(LD13 + 4B)”), and the treatment of “60 days-(LD13 + 4B)” always led to the greatest contents of non-enzymatic antioxidants.

### 3.7. Gene Expression

**Figure 8:**
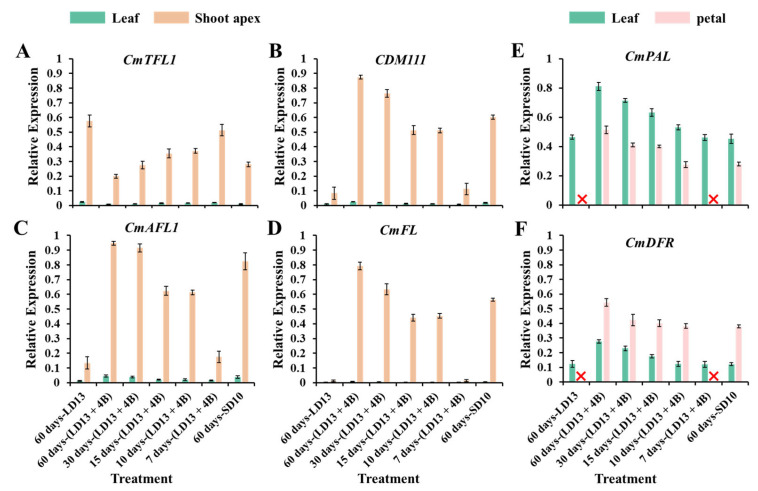
The tissue-specific expression patterns of flowering-related genes in leaves and shoot apices (**A**–**D**), and the tissue-specific expression patterns of antioxidant synthesis-related genes in leaves and petals (**E**,**F**), after 60 days of exposure to the photoperiodic light treatments. The top fourth true leaves, shoot apices, and fully blooming petals were harvested at 12:00 a.m. (4 h after white lights were turned on) for RNA extraction and RT-PCR. Data were averagely normalized against the expression of *CmACTIN* and *CmEF1α*. The maximum value in each experiment was set to “1”. The red cross (❌) demarcates the non-flowered treatments. Vertical bars indicate the means ± standard error of 6 biological replicates (*n* = 6), using RNA from separate plants. See Figure 1 and Table 1 for details of the experimental treatments.

To study the tissue-specific expression patterns of flowering-related genes in “Gaya Glory” in response to the proportion of S-BL days, the anti-florigenic *TFL1/CEN*-like gene (*CmTFL1*) and three well-characterized floral meristem identity genes *APETALA1* (*CDM111*), *FRUITFULL* (*CmAFL1*), and *LEAFY* (*CmFL*) were selected and analyzed by qRT-PCR in leaves and shoot apices, respectively (Figure 8A–D). After 60 days of exposure to the unusual photoperiodic light treatments, these flower-forming-related genes were all highly expressed in the shoot apices. However, extremely low or barely detectable expression was observed in leaves. The expression of the anti-florigenic gene *CmTFL1* was obviously higher in the non-flowered treatments “7 days-(LD13 + 4B)” and “60 days-LD13”, and showed the opposite proportional to the flowering capacity. However, these 3 floral meristem identity genes *CDM111*, *CmAFL1*, and *CmFL* were expressed inversely, the higher expression was observed in flowering-promoted treatments “60 days-(LD13 + 4B)” and “30 days-(LD13 + 4B)”, while significantly lower in non-flowered treatments “7 days-(LD13 + 4B)” and “60 days-LD13”.

The antioxidant ability of “Gaya Glory” plants strongly responded to the various proportions of S-BL days (Figure 6 and Figure 7). We also explored the tissue-specific expression patterns of the phenylalanine ammonia-lyase (PAL) gene (*CmPAL*) and the dihydroflavonol 4-reductase (DFR) gene (*CmDFR*) in the *Chrysanthemum morifolium* (Figure 8E,F). The non-flowered treatments “7 days-(LD13 + 4B)” and “60 days-LD13” were not included in the analysis of the expression of these two genes in petals. The *CmPAL* was more expressed in the leaves than in the petals, while the *CmDFR* was the opposite. Consistently, their expression trend increased with the proportion of S-BL days as did the trend in the antioxidant capacity. Still, the “60 days-(LD13 + 4B)” treatment always resulted in the best expression of these two antioxidant synthesis-related genes, both in the leaves and the petals.

### 3.8. Principal Component Analysis

**Figure 9:**
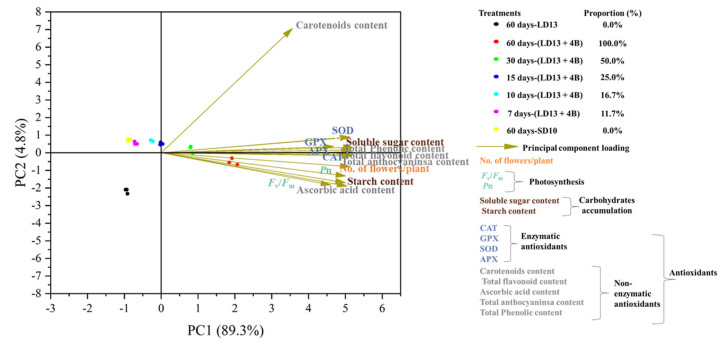
The principal component loading pot and scores of flowering-, photosynthesis-, carbohydrate-, or antioxidant-related parameters analyzed in “Gaya Glory” plants (*n* = 3) grown under different intervals of supplemental blue light, after 60 days of exposure to the photoperiodic light treatments. Proportion indicates the proportion of the number of days of supplemental blue light during the experimental duration. See Figure 1 and Table 1 for details of the experimental treatments.

The PCA of all analyzed parameters highlighted that the first two principal components (PCs) were related with eigenvalues higher than 1 and explained 94.1% of the total variance, with PC1 and PC2 accounting for 89.3% and 4.8%, respectively. The type of S-BL treatment contributed to the clear separation on PC1 (Figure 9). “60 days-(LD13 + 4B)” and “30 days-(LD13 + 4B)” treatments were concentrated on the positive side of PC1 and closed to the x-axis; the treatment of “15 days-(LD13 + 4B)” was concentrated in the y-axis; while the treatments of “10 days-(LD13 + 4B)”, “7 days-(LD13 + 4B)”, and “60 days-SD10” were located on the negative side of PC1. Especially the “60 days-LD13” treatment was in the lower left quadrant (Figure 9). PC1 was positively correlated to all the flowering-, photosynthesis-, carbohydrate-, or antioxidant-related parameters analyzed in “Gaya Glory” plants grown under different intervals of S-BL. Generally, the proportions of S-BL days were positively correlated with all these parameters. When the proportion of days of S-BL was over 50.0% (“30 days-(LD13 + 4B)”), the flowering, photosynthesis efficiency, carbohydrate accumulation, and antioxidant property were greatly promoted (Figure 9).

## 4. Discussion

### 4.1. The Chlorophyll Content, Photosynthetic Efficiency, Related Enzymatic Activities, and Accumulation of Nutrients in Response to Different Proportions of S-BL Days

Blue light has been shown to benefit the Chl content in numerous species, by advancing chloroplasts for an upgraded light capture capacity, thus improving the photosynthetic productivity [73,74]. “30 days-(LD13 + 4B)” and “60 days-(LD13 + 4B)” consistently increased the Chl content (Figure 3A,B). The higher Chl content might be attributed to the enhanced production of 5-aminolevulinic acid (ALA), the primary Chl substrate, which was boosted by blue light [75]. Furthermore, ALA encourages the conversion of Chl a to b, inhibiting the breakdown of the Chl content [76]. Since the value of Chl a/b governs the intensity of absorbed light, any increase in the Chl content or reduction in the Chl a/b will be beneficial to plants [77,78], which is consistent with our findings (Figure 3).

According to Shimazaki et al., stomata effectively opened in response to blue light [79]. Stomatal opening raises the intercellular CO_2_ content, which boosts the photosynthetic parameter values such as the net photosynthetic rate [80]. In our present study, when the proportion of S-BL days grew, the stomata-related metrics *G*s, *T*r, *C*i, etc. improved; 50.0% (“30 days-(LD13 + 4B)”) was a turning point in boosting those features, and the *P*n improved significantly over this proportion of S-BL days (Table 4). Chl fluorescence characteristics are essential markers in photosynthetic control because they immediately represent plant responses to changes in the light condition [81]. The increased photosynthetic capability is always associated with a high number of electrons passing via PSII [82]. Blue light increases the de novo production of the PSII reaction center complex’s core D1 (QB) protein [83], hence increasing photosynthesis. A previous study has found that blue light promotes high photosynthesis by increasing the *qP*, PSII, and photosynthetic electron transfer rate (ETR) [84]. As shown in Table 4, the Chl fluorescence indices increased as the days of S-BL increased, until their peaks appeared in “60 days-(LD13 + 4B)”.

Additionally, plants cultivated with blue light have higher contents of proteins, total soluble sugars, sucrose, starch, and greater dry weight, but shorter shoots compared to plants grown with white light [85,86]. Our results are also consistent with the above reports (Figure 2B and Figure 4, Table 3). In addition, the blue light not only actuates numerous compounds in starch combination, photosynthetic carbon absorption, photorespiration, and chlorophyll union pathways, but also prompts the amalgamation of RuBisCO [87,88], UDGPPase, and PEPC [89] to upgrade RuBisCO and nicotinamide adenine dinucleotide phosphate (NADP)-subordinate phosphoglycerate dehydrogenase [90]. The positive effects of blue light on photosynthesis- or carbohydrate synthesis-related enzymatic activities became significantly pronounced when the proportion of S-BL days was over 50.0% (“30 days-(LD13 + 4B)”) (Figure 5). Overall, suitable blue light supplementation maximizes the photosynthetic carbon assimilation efficiency and organic matter accumulation, providing sufficient energy supply for subsequent plant life processes, such as flowering.

### 4.2. The Branching and Flowering in Response to Different Proportions of S-BL Days

According to Gao et al. [91], the *CmTFL1* gene boosted secondary branching in *Arabidopsis* and axillary buds in *Chrysanthemum*, indicating that the high expression level of *CmTFL1* in the stem stimulated the establishment of lateral meristems. Similar effects have been observed in other species having homologous *TFL1* genes. The *LpTFL1* gene in *Lolium perenne* L. not only restored the *tfl1* mutant’s phenotype but also generated a large number of secondary branches and leaves with improved vegetative growth [92]. Furthermore, the *Arabidopsis AtTFL1*, *Prunus serotine PsTFL1*, and *Lotus japonicas LjCEN1* genes all increased the number of branches and leaves [93,94,95]. Hence, the *TFL1* gene has a conserved role in controlling branching and leafing. Moreover, the constitutive expression of *CsTFL1* was significantly delayed in flower production in *Chrysanthemum seticuspe* under SD circumstances. *CmTFL1* function was confirmed using five transgenic lines, and *CmTFL1* functionally influenced flower development in *Chrysanthemum morifolium* [66,96]. Similarly, transgenic *JcTFL1b*-RNAi *Jatropha* showed consistent early blooming [97]. In our current study, similar changing patterns in the number of leaves and branches were observed under different proportions of S-BL days: more branches or leaves but fewer flowers (Figure 2 and Table 3). The *CmTFL1* expression was typically higher in LD conditions and particularly high in treatments with a greater number of branches and leaves, especially in the non-flowered treatments “7 days-(LD13 + 4B)” and “60 days-LD13” (Figure 8A). Overall, the *CmTFL1* gene promotes branching and leafing while inhibiting blooming, making the plant bushier.

Furthermore, three chrysanthemum orthologues of *FT* in *Chrysanthemum seticuspe* were identified: *CsFTL1*, *CsFTL2*, and *CsFTL3*, and *CsFTL3* expression was recognized as a major regulator in chrysanthemum photoperiodic flowering [98]. The *CsFTL3* up-regulated floral-identity genes under flower-inducing SD circumstances to induce flowering events in the SAM [98]. Overexpression of *CsFTL3* in LD settings enhanced SDP chrysanthemum blooming, demonstrating that *CsFTL3* has the capacity to stimulate chrysanthemum flowering under photoperiod-unfavorable circumstances. Furthermore, rice (*Oryza sativa*), a facultative SDP, may blossom even in non-inductive LD circumstances. Two florigenic genes (*Hd3a* and *RFT1*) of rice are activated depending on the length of the day, and the RFT1 protein is proposed as the LD Florien [99]. *CmFTL1* in chrysanthemum may operate as an LD florigenic gene, comparable to *RFT1* in rice. Under photoperiod-unfavorable conditions, it may be anticipated that residual *CmFTL3* and enhanced *CmFTL1* eventually cause flowering, which was also proved by our pervious study exploring the LD florigenic-like gene *CmFTL1* involved in photoperiodic flowering in SDP chrysanthemum [13]. That might be one of the regions to induce more flowers in “60 days-(LD13 + 4B)” and “30 days-(LD13 + 4B)” treatments (Figure 2 and Table 3).

Numerous studies have found that sugars and their metabolites regulate blooming in plants. Arabidopsis, for example, may blossom in full darkness due to a lack of sucrose at the plant’s tip. In vitro studies on tomatoes have also revealed that appropriate levels of sucrose and nitrogen stimulate blooming. In addition, sugar can control plant blooming by activating and suppressing the expression of certain genes. In late flowering mutants, for example, 1% (*w*/*v*) sucrose may activate *LFY* (*LEAFY*) expression and stimulate plant blooming via the LFY factor [100,101,102]. Moreover, the flowering-improved treatments “60 days-(LD13 + 4B)” and “30 days-(LD13 + 4B)” also resulted in the higher expression of florigenic genes (like *CDM111*, *CmAFL1*, and *CmFL*) (Figure 8B–D). Furthermore, the contents of total soluble sugars and starch were also significantly higher in these treatments (Figure 4A,B).

### 4.3. The Antioxidant Property and the Expression of Antioxidant Synthesis-Related Genes in Response to Different Proportions of S-BL Days

In general, enzymatic and non-enzymatic antioxidant defense mechanisms interact together synergistically to keep ROS levels balanced and avoid oxidative stresses. It has been claimed that the light quality, particularly blue light, an efficient activator in stimulating antioxidant systems, has a considerable influence on the antioxidant capacity in many plant species [103,104]. Furthermore, ALA as a Chl substrate is positively connected with the antioxidant capacity [105], which may be enhanced by blue light [75], suggesting that Chl has the potential to eliminate excess ROS in plants. From our results, the activities of SOD, CAT, APX, and GPX improved as the proportion of S-BL days increased, especially when the proportion was equal to or greater than 50.0% (“30 days-(LD13 + 4B)”) (Figure 6). Consistent with the above references, the Chl content was markedly increased when the proportion of S-BL days was over 50.0% (“30 days-(LD13 + 4B)”) (Figure 3A,B), which laid the foundation for the antioxidant capacity increase. Blue light modulates plants in response to any biotically or abiotically stressful environments, indicating the ability of blue light to activate the antioxidant system [24].

The non-enzymatic antioxidant capacity of chrysanthemum leaves cultivated under different proportions of S-BL days was also investigated. Carotenoids, tocopherol, flavonoids, ascorbate, and phenolic compounds are antioxidants found in plants that play key roles in protection against photo-oxidative damage [18,22]. The number and content of phenolic compounds in growing edible fruits are greatly impacted by the light quality, according to Luthria et al. [106]. Furthermore, Johkan et al. observed that S-BL radiation enhanced the level of phenolic compounds in red leaf lettuce [107]. Our results are in agreement with data published by Johkan et al. [107], in which the content of polyphenols and antioxidant activity were shown to be greatly increased in chrysanthemum leaves treated with higher proportions of S-BL days, and the 50.0% (“30 days-(LD13 + 4B)”), especially the 100.0% (“60 days-(LD13 + 4B)”) resulted in the greater production of these non-enzymatic antioxidants (Figure 7). Taken together, our results clearly indicated that the contents of proline and total phenolic compounds in leaves of chrysanthemums were considerably influenced by the B LEDs. In particular, the proportion of S-BL days over 50.0% dramatically increased the contents of proline and phenolic compounds in vegetative tissues in plants.

Additionally, blue light not only directly regulates the production of antioxidants but also indirectly controls their synthesis by mediating the expression of antioxidant synthesis-related genes. In our study, two genes for anthocyanin synthesis-related enzymes, *CmPAL* and *CmDFR*, were tested. Phenylalanine ammonia-lyase (PAL) is the 1st rate-limiting enzyme of the phenylalanine metabolic pathway which can be involved in plant immune response, and is a key enzyme in the process of plant stress responses [108]. Its enzymatic activity and gene transcription are influenced by various environmental factors, especially related to the stress response involving phenylalanine [109]. It is speculated that the high expression of *PAL* may be related to the synthesis of anthocyanins [110]. Dihydroflavonol 4-reductase (DFR) is a key enzyme in the process of anthocyanin synthesis [111]. As one of the flavonoids, anthocyanin not only directly regulates the color of flowers, fruits, and seeds of plants, but also has certain pharmacological effects (antitumor, hypoglycemic, etc.) [112]. The tissue-specific expression patterns of these two genes in the *Chrysanthemum morifolium* trend increased with the proportion of S-BL days as did the pattern in the antioxidant capacity. Still, the “60 days-(LD13 + 4B)” treatment always resulted in the best expression of these two antioxidant synthesis-related genes in both leaves and petals (Figure 8E,F). Combined, the results of the present study indicated that blue LED supplementation and the proportions of S-BL days had positive effects on the action of antioxidant capacity in chrysanthemums.

### 4.4. The Positive Interactive Relations among the Blue Light-Mediated-Photosynthetic Carbon Assimilation, -Antioxidant Ability, and -Flowering

In the current study, the principal component analysis (PCA) indicated that the proportion of S-BL days positively regulated flowering, photosynthesis, carbohydrate accumulation, and antioxidant production. When the proportion of days of S-BL was over 50.0% (“30 days-(LD13 + 4B)”), these parameters were significantly improved (Figure 9). According to several findings, blue light affects photoperiodic blooming in chrysanthemums via the co-regulation of photosynthetic carbon assimilation and differential photoreceptor-mediated mechanisms [13]. Meanwhile, sugar metabolism has a significant role in both ROS generation and scavenging mechanisms [33,34,35]. Thus, the sugar-involved flowering and sugar-related antioxidant abilities can be controlled by blue light-affected photosynthetic efficiency and carbon assimilation. Furthermore, one investigation discovered that the non-enzymatic cell reinforcement ascorbic corrosive capabilities co-calculate the biosynthesis of GA and ABA, impacting the endogenous level as well as the motioning of these plant chemicals, and subsequently influencing formative blossoming and senescence in a photoperiod-or potentially circadian cadence subordinate way [36]. However, how ascorbic acid or other antioxidants control flowering requires further investigation.

## 5. Conclusions

In conclusion, the proportion of S-BL days positively regulated flowering, photosynthesis, carbohydrate accumulation, and antioxidant production. According to our current findings, (1) the number of flowers increased and the flower buds appeared earlier as the proportion of S-BL days increased; (2) the proportion of initial, pivotal, and optimal flowering is 16.7% (“10 days-(LD13 + 4B)”), 50.0% (“30 days-(LD13 + 4B)”), and 100.0% (“60 days-(LD13 + 4B)”), respectively; (3) the 50.0% (“30 days-(LD13 + 4B)”) appears to be a turning point for the change in the physiological traits until peaks appeared in 100.0% (“60 days-(LD13 + 4B)”). In aggregate, the pivotal and optimal proportions of S-BL days to reconcile the relationship among flowering, photosynthetic carbon assimilation, and antioxidant ability is 50.0% (“30 days-(LD13 + 4B)”) and 100.0% (“60 days-(LD13 + 4B)”), respectively. We hope our study could inspire researchers and growers to choose the appropriate LED lighting options for flowering control, phytochemical contents, nutritional value, etc. This study also provides support for the use of blue light in horticulture. Studies continue to elucidate the precise role of blue light and antioxidant abilities in regulating flowering.

## Figures and Tables

**Table 1:** Description and abbreviation of the photoperiodic light treatments.

Description	Abbreviation	Proportion ^2^ (%)
1 day (LD13) × 60 ^1^ cycles	60 days-LD13	0.0
[0 day (LD13) + 1 day (LD13 + 4B)] × 60 cycles	60 days-(LD13 + 4B)	100.0
[1 day (LD13) + 1 day (LD13 + 4B)] × 30 cycles	30 days-(LD13 + 4B)	50.0
[3 days (LD13) + 1 day (LD13 + 4B)] × 15 cycles	15 days-(LD13 + 4B)	25.0
[5 days (LD13) + 1 day (LD13 + 4B)] × 10 cycles	10 days-(LD13 + 4B)	16.7
[7 days (LD13) + 1 day (LD13 + 4B)] × 7 cycles + 4 days (LD13)	7 days-(LD13 + 4B)	11.7
1 day (SD10) × 60 cycles	60 days-SD10	0.0

^1^ 60 days, the total experimental duration. ^2^ The proportion of the number of days supplemental blue light was provided during the total experimental duration. The numbers in a green color represent the different intervals in which the supplemental blue light was provided; the numbers in a red color represent the days that blue light is used during the total experimental duration. Control groups: “60 days-LD13” and “60 days-SD10”; experimental groups: “60 days-(LD13 + 4B)”, “30 days-(LD13 + 4B)”, “15 days-(LD13 + 4B)”, “10 days-(LD13 + 4B)”, and “7 days-(LD13 + 4B)”.

**Table 2:** Primers and PCR conditions used to quantify the gene expression levels.

Reference gene	Name	Accession number	Forward primer (5′ to 3′)	Reverse primer (5′ to 3′)
	*CmACTIN* [64]	AB205087	GATGACGCAGATCATGTTCG	AGCATGTGGAAGTGCATACC
	*CmEF1α* [65]	AB548817	CTTGTTGCTTGATGACTGTGG	CTTGTTGCTTGATGACTGTGG
Flowering-related gene	*CmTFL1* [66]	AB839767	CCATCATCAAGGCACAATTTCA	TTTCCCTTTGGCAGTTGAAGAA
	*CDM111* [67]	AY173054	GGTCTCAAGAATATTCGCAC	TCATTAGTCATCCCATCAGC
	*CmAFL1* [68]	AB451218	CAAGCTCAACCATCAATAGTC	TGCAGCACATGAACGAGTAG
	*CmFL* [68]	AB451217	CATTGATGCCATATTTAACTC	ACACGGATCATTCATTGTATA
	PCR conditions		PCR was performed with an initial denaturing step at 95 °C for 5 min, followed by 40 cycles at 95 °C for 5 s, 60 °C for 20 s, 72 °C for 30 s, and 72 °C for 10 min to final extension. The fluorescence was quantified after the incubation at 72 °C and repeated three times.	
Antioxidant synthesis-related gene	*CmPAL* [69]	JF806362	TACATTTTGGACGGGAGTGA	AGCGTATCGGTCTTGTTTGG
	*CmDFR* [70]	GU324979	GACATTATGGAAGGCGGATT	GTGGCAACATGAAACACTC
	PCR conditions		30-s pre-denaturation at 95 °C followed by 5 s at 95 °C, 30 s at 60 °C, 40 cycles; the melting curves were 15 s at 95 °C, 60 s at 60 °C, and 15 s at 95 °C. The fluorescence signal was collected once for every 0.3 °C increase and repeated three times.	

**Table 3:** The growth parameters of “Gaya Glory” under different intervals of supplemental blue light, after 60 days of exposure to the photoperiodic light treatments.

Treatment	Shoot			
	Shoot length (cm)	No. of branches/Plant	Dry weight (g)	
60 days-LD13	14.8 ± 0.53 ab ^1^	16.3 ± 1.03 a	3.72 ± 0.05 bc	
60 days-(LD13 + 4B)	15.8 ± 0.41 a	11.4 ± 0.97 b	6.50 ± 0.10 a	
30 days-(LD13 + 4B)	15.7 ± 0.47 a	11.3 ± 1.01 b	5.81 ± 0.09 ab	
15 days-(LD13 + 4B)	15.5 ± 0.50 a	14.4 ± 1.12 ab	4.63 ± 0.07 b	
10 days-(LD13 + 4B)	13.5 ± 0.45 b	14.8 ± 1.07 ab	4.24 ± 0.06 b	
7 days-(LD13 + 4B)	13.2 ± 0.43 b	16.2 ± 1.00 a	3.92 ± 0.05 bc	
60 days-SD10	9.3 ± 0.38 c	7.7 ± 0.96 c	1.80 ± 0.05 c	
Treatment	Leaf		Flower	
	No. of leaves/Plant	Leaf area ^2^ (cm^2^)	DVB ^3^ (day)	No. of flowers/Plant
60 days-LD13	337.2 ± 10.98 a	3.8 ± 0.09 bc	-	0.0 ± 0.00 e
60 days-(LD13 + 4B)	225.4 ± 6.97 c	6.1 ± 0.11 a	29.7 ± 1.74 c	43.9 ± 5.76 a
30 days-(LD13 + 4B)	230.0 ± 6.24 c	5.2 ± 0.08 ab	34.2 ± 2.11 b	25.7 ± 3.24 b
15 days-(LD13 + 4B)	276.7 ± 9.36 b	4.5 ± 0.13 b	48.6 ± 2.96 ab	8.4 ± 1.06 d
10 days-(LD13 + 4B)	280.4 ± 7.01 b	3.8 ± 0.17 bc	51.9 ± 2.03 a	5.6 ± 1.12 d
7 days-(LD13 + 4B)	301.6 ± 7.64 ab	3.8 ± 0.20 bc	-	0.0 ± 0.00 e
60 days-SD10	181.2 ± 8.52 d	2.9 ± 0.10 c	26.2 ± 1.57 cd	17.8 ± 3.73 c

^1^ Mean separation within columns by the Duncan’s multiple range test at *p* ≤ 0.05 and the values are average ± standard error (*n* = 6). ^2^ Area of the fourth mature leaf from the shoot tip. ^3^ The days to visible flower buds in each treatment were determined by counting the number of days from initiating the light treatment to the date when the first flower bud appeared.

**Table 4:** The photosynthetic and chlorophyll fluorescence characteristics of “Gaya Glory” under different intervals of supplemental blue light, after 60 days of exposure to the photoperiodic light treatments.

Treatment	*P*n ^1^ (μmol CO_2_ m^−^2·s^−1^)	*T*r ^2^ (mmol H_2_O m^−^2·s^−1^)	*G*s ^3^ (mol H_2_O m^−^2·s^−1^)	*C*i ^4^ (μmol CO_2_ mol^−1^)	*F*v/*F*m ^5^	*F*v′/*F*m′ ^6^	*qP* ^7^
60 days-LD13	12.02 ± 0.06 bc ^8^	1.52 ± 0.01 c	0.50 ± 0.007 c	384.6 ± 2.24 c	0.81 ± 0.004 ab	0.43 ± 0.001 bc	0.45 ± 0.002 bc
60 days-(LD13 + 4B)	18.03 ± 0.07 a	2.12 ± 0.02 a	0.83 ± 0.009 a	462.7 ± 3.97 a	0.86 ± 0.003 a	0.68 ± 0.004 a	0.67 ± 0.004 a
30 days-(LD13 + 4B)	16.31 ± 0.07 ab	1.91 ± 0.04 ab	0.74 ± 0.006 ab	443.1 ± 3.09 ab	0.85 ± 0.006 a	0.62 ± 0.006 ab	0.60 ± 0.003 ab
15 days-(LD13 + 4B)	14.11 ± 0.04 b	1.80 ± 0.02 b	0.65 ± 0.004 b	422.7 ± 4.01 b	0.82 ± 0.007 ab	0.51 ± 0.002 b	0.53 ± 0.007 b
10 days-(LD13 + 4B)	14.08 ± 0.06 b	1.65 ± 0.03 bc	0.58 ± 0.009 bc	407.1 ± 2.36 bc	0.81 ± 0.005 ab	0.51 ± 0.003 b	0.51 ± 0.008 b
7 days-(LD13 + 4B)	12.07 ± 0.08 bc	1.63 ± 0.04 bc	0.57 ± 0.005 bc	404.9 ± 1.28 bc	0.80 ± 0.004 ab	0.46 ± 0.005 bc	0.47 ± 0.002 bc
60 days-SD10	10.52 ± 0.06 c	1.49 ± 0.05 c	0.49 ± 0.007 c	378.8 ± 3.79 c	0.80 ± 0.007 ab	0.37 ± 0.003 c	0.40 ± 0.001 c

^1^ Net photosynthetic rate (*P*n); ^2^ Transpiration rate (*T*r); ^3^ Stomatal conductance (*G*s); ^4^ Intercellular CO_2_ concentration (*C*i); ^5^ Maximal PSII quantum yield (*F*v/*F*m); ^6^ Photochemical efficiency of PSII (*F*v′/*F*m′); ^7^ Photochemical quenching coefficient (*qP*); ^8^ Mean separation within columns by the Duncan’s multiple range test at *p* ≤ 0.05 and the values are average ± standard error (*n* = 6).

## Data Availability

Data sharing is not applicable to this article.

## References

[1] Thomas B., Vince-Prue D. (1997). Photoperiodic control of flower initiation: Some general principles. Photoperiodism in Plants.

[2] Kozai T. (2018). Current status of plant factories with artificial lighting (PFALs) and smart PFALs. Smart Plant Factory.

[3] Bantis F., Smirnakou S., Ouzounis T., Koukounaras A., Ntagkas N., Radoglou K. (2018). Current status and recent achievements in the field of horticulture with the use of light-emitting diodes (LEDs). Sci. Hortic..

[4] Kigel J., Cosgrove D.J. (1991). Photoinhibition of stem elongation by blue and red light: Effects on hydraulic and cell wall properties. Plant Physiol..

[5] CASAL J.J., SMITH H. (1989). Effects of blue light pretreatments on internode extension growth in mustard seedlings after the transition to darkness: Analysis of the interaction with phytochrome. J. Exp. Bot..

[6] Laskowski M.J., Briggs W.R. (1989). Regulation of pea epicotyl elongation by blue light: Fluence-response relationships and growth distribution. Plant Physiol..

[7] Warpeha K.M., Kaufman L.S. (1989). Blue-light regulation of epicotyl elongation in *Pisum sativum*. Plant Physiol..

[8] Schneider M., Borthwick H., Hendricks S. (1967). Effects of radiation on flowering of *Hyoscyamus niger*. Am. J. Bot..

[9] Bagnall D.J., King R.W., Hangarter R.P. (1996). Blue-light promotion of flowering is absent in *hy4* mutants of *Arabidopsis*. Planta.

[10] Park Y., Runkle E.S. (2018). Spectral effects of light-emitting diodes on plant growth, visual color quality, and photosynthetic photon efficacy: White versus blue plus red radiation. PLoS ONE.

[11] Runkle E.S., Heins R.D. (2001). Specific functions of red, far red, and blue light in flowering and stem extension of long-day plants. J. Am. Soc. Hortic. Sci..

[12] Park Y.G., Jeong B.R. (2020). How supplementary or night-interrupting low-intensity blue light affects the flower induction in chrysanthemum, a qualitative short-day plant. Plants.

[13] Yang J., Song J., Jeong B.R. (2022). The flowering of SDP chrysanthemum in response to intensity of supplemental or night-interruptional blue light is modulated by both photosynthetic carbon assimilation and photoreceptor-mediated regulation. Front. Plant Sci..

[14] Yang J., Song J., Jeong B.R. (2022). Low-intensity blue light supplemented during photoperiod in controlled environment induces flowering and antioxidant production in kalanchoe. Antioxidants.

[15] Koch K. (2004). Sucrose metabolism: Regulatory mechanisms and pivotal roles in sugar sensing and plant development. Curr. Opin. Plant Biol..

[16] Gangadhar B.H., Mishra R.K., Pandian G., Park S.W. (2012). Comparative study of color, pungency, and biochemical composition in chili pepper (*Capsicum annuum*) under different light-emitting diode treatments. HortScience.

[17] Urbonavičiūtė A., Pinho P., Samuolienė G., Duchovskis P., Vitta P., Stonkus A., Tamulaitis G., Žukauskas A., Halonen L. (2007). Effect of short-wavelength light on lettuce growth and nutritional quality. Sodininkystė Daržininkystė.

[18] Samuolienė G., Brazaitytė A., Urbonavičiūtė A., Šabajevienė G., Duchovskis P. (2010). The effect of red and blue light component on the growth and development of frigo strawberries. Zemdirbyste.

[19] Avercheva O., Berkovich Y.A., Smolyanina S., Bassarskaya E., Pogosyan S., Ptushenko V., Erokhin A., Zhigalova T. (2014). Biochemical, photosynthetic and productive parameters of chinese cabbage grown under blue–red LED assembly designed for space agriculture. Adv. Space Res..

[20] Chen X.L., Guo W.Z., Xue X.Z., Wang L.C., Qiao X.J. (2014). Growth and quality responses of ‘green oak leaf’ lettuce as affected by monochromic or mixed radiation provided by fluorescent lamp (FL) and light-emitting diode (LED). Sci. Hortic..

[21] Torres M.A., Jones J.D., Dangl J.L. (2006). Reactive oxygen species signaling in response to pathogens. Plant Physiol..

[22] Ashry N.A., Mohamed H.I. (2011). Impact of secondary metabolites and related enzymes in flax resistance and or susceptibility to powdery mildew. World J. Agric. Sci.

[23] Sharma P., Jha A.B., Dubey R.S., Pessarakli M. (2012). Reactive oxygen species, oxidative damage, and antioxidative defense mechanism in plants under stressful conditions. J. Bot..

[24] Kim K., Kook H., Jang Y., Lee W., Kamala-Kannan S., Chae J., Lee K. (2013). The effect of blue-light-emitting diodes on antioxidant properties and resistance to *Botrytis cinerea* in tomato. J. Plant Pathol. Microbiol..

[25] Samuolienė G., Brazaitytė A., Jankauskienė J., Viršilė A., Sirtautas R., Novičkovas A., Sakalauskienė S., Sakalauskaitė J., Duchovskis P. (2013). Led irradiance level affects growth and nutritional quality of *Brassica* microgreens. Cent. Eur. J. Biol..

[26] Son K.H., Oh M.M. (2013). Leaf shape, growth, and antioxidant phenolic compounds of two lettuce cultivars grown under various combinations of blue and red light-emitting diodes. HortScience.

[27] Ren J., Guo S., Xu C., Yang C., Ai W., Tang Y., Qin L. (2014). Effects of different carbon dioxide and LED lighting levels on the anti-oxidative capabilities of *Gynura bicolor DC*. Adv. Space Res..

[28] Ouzounis T., Fretté X., Rosenqvist E., Ottosen C.-O. (2014). Spectral effects of supplementary lighting on the secondary metabolites in roses, chrysanthemums, and campanulas. J. Plant Physiol..

[29] Kim E.Y., Park S., Park B.J., Lee Y., Oh M.M. (2014). Growth and antioxidant phenolic compounds in cherry tomato seedlings grown under monochromatic light-emitting diodes. Hortic. Environ. Biotechnol..

[30] Wu M.C., Hou C.Y., Jiang C.M., Wang Y.T., Wang C.Y., Chen H.H., Chang H.M. (2007). A novel approach of LED light radiation improves the antioxidant activity of pea seedlings. Food Chem..

[31] Walters R.G., Ibrahim D.G., Horton P., Kruger N.J. (2004). A mutant of *Arabidopsis* lacking the triose-phosphate/phosphate translocator reveals metabolic regulation of starch breakdown in the light. Plant Physiol..

[32] Eckardt N.A., Snyder G.W., Portis Jr A.R., Ogren W.L. (1997). Growth and photosynthesis under high and low irradiance of *Arabidopsis thaliana* antisense mutants with reduced Ribulose-1, 5-Bisphosphate Carboxylase/Oxygenase Activase content. Plant Physiol..

[33] Sami F., Yusuf M., Faizan M., Faraz A., Hayat S. (2016). Role of sugars under abiotic stress. Plant Physiol. Biochem..

[34] Van den Ende W., Peshev D. (2013). Sugars as antioxidants in plants. Crop Improvement under Adverse Conditions.

[35] Keunen E., Peshev D., Vangronsveld J., Van Den Ende W., Cuypers A. (2013). Plant sugars are crucial players in the oxidative challenge during abiotic stress: Extending the traditional concept. Plant Cell Environ..

[36] Barth C., De Tullio M., Conklin P.L. (2006). The role of ascorbic acid in the control of flowering time and the onset of senescence. J. Exp. Bot..

[37] Lichtenthaler H.K., Buschmann C. (2001). Chlorophylls and carotenoids: Measurement and characterization by UV-VIS spectroscopy. Curr. Protoc. Food Anal. Chem..

[38] Genty B., Briantais J.M., Baker N.R. (1989). The relationship between the quantum yield of photosynthetic electron transport and quenching of chlorophyll fluorescence. BBA-Gen. Subj..

[39] Roháček K. (2002). Chlorophyll fluorescence parameters: The definitions, photosynthetic meaning, and mutual relationships. Photosynthetica.

[40] Loewus F.A. (1952). Improvement in anthrone method for determination of carbohydrates. Anal. Chem..

[41] Yemm E., Willis A. (1954). The estimation of carbohydrates in plant extracts by anthrone. Biochem. J..

[42] Bradford M.M. (1976). A rapid and sensitive method for the quantitation of microgram quantities of protein utilizing the principle of protein-dye binding. Anal. Biochem..

[43] Doehlert D.C., Kuo T.M., Felker F.C. (1988). Enzymes of sucrose and hexose metabolism in developing kernels of two inbreds of maize. Plant Physiol..

[44] Liang J.S., Cao X., Xu S., Zhu Q., Song P. (1994). Studies on the relationship between the grain sink strength and its starch accumulation in rice (*O. sativa*). Acta Agron. Sin.

[45] Yang L.T., Chen L.S., Peng H.Y., Guo P., Wang P., Ma C.L. (2012). Organic acid metabolism in *Citrus grandis* leaves and roots is differently affected by nitric oxide and aluminum interactions. Sci. Hortic..

[46] Feng L., Raza M.A., Li Z., Chen Y., Khalid M.H.B., Du J., Liu W., Wu X., Song C., Yu L. (2019). The influence of light intensity and leaf movement on photosynthesis characteristics and carbon balance of soybean. Front. Plant Sci..

[47] Aebi H. (1974). Catalase. Methods of Enzymatic Analysis.

[48] Castillo F.J., Penel C., Greppin H. (1984). Peroxidase release induced by ozone in sedum album leaves: Involvement of Ca^2+^. Plant Physiol..

[49] Becana M., Aparicio-Tejo P., Irigoyen J.J., Sanchez-Diaz M. (1986). Some enzymes of hydrogen peroxide metabolism in leaves and root nodules of *Medicago sativa*. Plant Physiol..

[50] Nakano Y., Asada K. (1981). Hydrogen peroxide is scavenged by ascorbate-specific peroxidase in spinach chloroplasts. Plant Cell Physiol..

[51] Andre C.M., Ghislain M., Bertin P., Oufir M., del Rosario Herrera M., Hoffmann L., Hausman J.-F., Larondelle Y., Evers D. (2007). Andean potato cultivars (*Solanum tuberosum* L.) as a source of antioxidant and mineral micronutrients. J. Agr. Food Chem..

[52] Rodriguez-Amaya D.B. (2001). A Guide to Carotenoid Analysis in Foods.

[53] Awika J.M., Rooney L.W., Waniska R.D. (2005). Anthocyanins from black sorghum and their antioxidant properties. Food Chem..

[54] Nielsen I.L.F., Haren G.R., Magnussen E.L., Dragsted L.O., Rasmussen S.E. (2003). Quantification of anthocyanins in commercial black currant juices by simple high-performance liquid chromatography. Investigation of their pH stability and antioxidative potency. J. Agric. Food Chem..

[55] Schultze M. (1947). Methods of vitamin assay. Prepared and edited by the association of vitamin chemists. J. Phys. Chem..

[56] Perva-Uzunalić A., Škerget M., Knez Ž., Weinreich B., Otto F., Grüner S. (2006). Extraction of active ingredients from green tea (*Camellia sinensis*): Extraction efficiency of major catechins and caffeine. Food Chem..

[57] Proestos C., Boziaris I., Nychas G.-J., Komaitis M. (2006). Analysis of flavonoids and phenolic acids in Greek aromatic plants: Investigation of their antioxidant capacity and antimicrobial activity. Food Chem..

[58] Siddhuraju P., Becker K. (2003). Antioxidant properties of various solvent extracts of total phenolic constituents from three different agroclimatic origins of drumstick tree (*Moringa oleifera* Lam.) leaves. J. Agric. Food Chem..

[59] Kumaran A. (2006). Antioxidant and free radical scavenging activity of an aqueous extract of *Coleus aromaticus*. Food Chem..

[60] Atanassova M., Georgieva S., Ivancheva K. (2011). Total phenolic and total flavonoid contents, antioxidant capacity and biological contaminants in medicinal herbs. J. Univ. Chem. Technol. Metall..

[61] Shahidi F., Naczk M., Griffiths W. (1996). Food phenolics: Sources, chemistry, effects, applications. Trends Food Sci. Technol..

[62] Waterhouse A.L. (2002). Wine phenolics. Ann. N. Y. Acad. Sci..

[63] Livak K.J., Schmittgen T.D. (2001). Analysis of relative gene expression data using real-time quantitative PCR and the 2^−△△ct^ method. Methods.

[64] Higuchi Y., Sumitomo K., Oda A., Shimizu H., Hisamatsu T. (2012). Day light quality affects the night-break response in the short-day plant chrysanthemum, suggesting differential phytochrome-mediated regulation of flowering. J. Plant Physiol..

[65] Gu C., Chen S., Liu Z., Shan H., Luo H., Guan Z., Chen F. (2011). Reference gene selection for quantitative real-time PCR in chrysanthemum subjected to biotic and abiotic stress. Mol. Biotechnol..

[66] Higuchi Y., Narumi T., Oda A., Nakano Y., Sumitomo K., Fukai S., Hisamatsu T. (2013). The gated induction system of a systemic floral inhibitor, antiflorigen, determines obligate short-day flowering in chrysanthemums. Proc. Natl. Acad. Sci. USA.

[67] Shchennikova A.V., Shulga O.A., Immink R., Skryabin K.G., Angenent G.C. (2004). Identification and characterization of four chrysanthemum MADS-box genes, belonging to the *APETALA1*/*FRUITFULL* and *SEPALLATA3* subfamilies. Plant Physiol..

[68] Li T., Niki T., Nishijima T., Douzono M., Koshioka M., Hisamatsu T. (2009). Roles of *CmFL*, *CmAFL1*, and *CmSOC1* in the transition from vegetative to reproductive growth in *Chrysanthemum morifolium* Ramat. J. Hortic. Sci. Biotechnol..

[69] An C., Zhang Y., Zhang W.W., Zhang T., Jiang J., Chen F. (2019). Cloning and expression analysis of *CmPAL* gene in *Chrysanthemum morifolium*. J. Nanjing Agric. Univ..

[70] Chen L., Wang T., Guo Q.S., Zhang X.M., Song L.S. (2016). Cloning of *DFR* gene and its expression characteristics in flowers of *Chrysanthemum morifolium*. Zhong Cao Yao (Chin. Tradit. Herb. Drugs).

[71] Ciarmiello L.F., Piccirillo P., Carillo P., De Luca A., Woodrow P. (2015). Determination of the genetic relatedness of fig (*Ficus carica* L.) accessions using rapid fingerprint and their agro-morphological characterization. S. Afr. J. Bot..

[72] Ferchichi S., Hessini K., Dell’Aversana E., D’Amelia L., Woodrow P., Ciarmiello L.F., Fuggi A., Carillo P. (2018). *Hordeum vulgare* and *Hordeum maritimum* respond to extended salinity stress displaying different temporal accumulation pattern of metabolites. Funct. Plant Biol..

[73] Snowden M.C., Cope K.R., Bugbee B. (2016). Sensitivity of seven diverse species to blue and green light: Interactions with photon flux. PLoS ONE.

[74] Klem K., Gargallo-Garriga A., Rattanapichai W., Oravec M., Holub P., Veselá B., Sardans J., Peñuelas J., Urban O. (2019). Distinct morphological, physiological, and biochemical responses to light quality in barley leaves and roots. Front. Plant Sci..

[75] Liu X., Li Y., Zhong S. (2017). Interplay between light and plant hormones in the control of *Arabidopsis* seedling chlorophyll biosynthesis. Front. Plant Sci..

[76] Tanaka Y., Tanaka A., Tsuji H. (1993). Effects of 5-Aminolevulinic Acid on the accumulation of chlorophyll b and apoproteins of the light-harvesting chlorophyll a/b-protein complex of photosystem II. Plant Cell Physiol..

[77] Melis A., Harvey G. (1981). Regulation of photosystem stoichiometry, chlorophyll a and chlorophyll b content and relation to chloroplast ultrastructure. Biochim. Biophys. Acta (BBA)-Bioenerg..

[78] Tanaka A., Ito H., Tanaka R., Tanaka N.K., Yoshida K., Okada K. (1998). Chlorophyll *a* oxygenase (*CAO*) is involved in chlorophyll *b* formation from chlorophyll *a*. Proc. Natl. Acad. Sci. USA.

[79] Shimazaki K.I., Doi M., Assmann S.M., Kinoshita T. (2007). Light regulation of stomatal movement. Annu. Rev. Plant Biol..

[80] Collatz G.J., Ball J.T., Grivet C., Berry J.A. (1991). Physiological and environmental regulation of stomatal conductance, photosynthesis and transpiration: A model that includes a laminar boundary layer. Agric. For. Meteorol..

[81] Dai Y., Shen Z., Liu Y., Wang L., Hannaway D., Lu H. (2009). Effects of shade treatments on the photosynthetic capacity, chlorophyll fluorescence, and chlorophyll content of *Tetrastigma hemsleyanum* Diels et Gilg. Environ. Exp. Bot..

[82] Yao X., Li C., Li S., Zhu Q., Zhang H., Wang H., Yu C., St Martin S.K., Xie F. (2017). Effect of shade on leaf photosynthetic capacity, light-intercepting, electron transfer and energy distribution of soybeans. Plant Growth Regul..

[83] Richter G., Wessel K. (1985). Red light inhibits blue light-induced chloroplast development in cultured plant cells at the mRNA level. Plant Mol. Biol..

[84] Huang D., Wu L., Chen J., Dong L. (2011). Morphological plasticity, photosynthesis and chlorophyll fluorescence of *Athyrium pachyphlebium* at different shade levels. Photosynthetica.

[85] Wang H., Gu M., Cui J., Shi K., Zhou Y., Yu J. (2009). Effects of light quality on CO_2_ assimilation, chlorophyll-fluorescence quenching, expression of calvin cycle genes and carbohydrate accumulation in *Cucumis sativus*. J. Photochem. Photobiol. B Biol..

[86] Xu D., Gao W., Ruan J. (2015). Effects of light quality on plant growth and development. Plant Physiol. J..

[87] Hundrieser J., Richter G. (1982). Blue light-induced synthesis of ribulosebisphosphate carboxylase in cultured plant cells. Plant Cell Rep..

[88] Roscher E., Zetsche K. (1986). The effects of light quality and intensity on the synthesis of ribulose-1, 5-bisphosphate carboxylase and its mrnas in the green alga *Chlorogonium elongatum*. Planta.

[89] Kamiya A., Miyachi S. (1975). Blue light-induced formation of phosphoenolpyruvate carboxylase in colorless *Chlorella* mutant cells. Plant Cell Physiol..

[90] Conradt W., Ruyters G. (1980). Blue light-effects on enzymes of the carbohydrate metabolism in *Chlorella* 2. Glyceraldehyde 3-phosphate dehydrogenase (NADP-dependent). The Blue Light Syndrome.

[91] Gao Y., Gao Y., Wu Z., Bu X., Fan M., Zhang Q. (2019). Characterization of *TERMINAL FLOWER 1* homologs *CmTFL1c* gene from *Chrysanthemum morifolium*. Plant Mol. Biol..

[92] Jensen C.S., Salchert K., Nielsen K.K. (2001). A *Terminal Flower1*-Like gene from perennial ryegrass involved in floral transition and axillary meristem identity. Plant Physiol..

[93] Ratcliffe O.J., Amaya I., Vincent C.A., Rothstein S., Carpenter R., Coen E.S., Bradley D.J. (1998). A common mechanism controls the life cycle and architecture of plants. Development.

[94] Wang Y., Pijut P.M. (2013). Isolation and characterization of a *TERMINAL FLOWER 1* homolog from *Prunus serotina* Ehrh. Tree Physiol..

[95] Guo X., Zhao Z., Chen J., Hu X., Luo D. (2006). A putative *CENTRORADIALIS/TERMINAL FLOWER 1-like* gene, *Ljcen1*, plays a role in phase transition in *Lotus japonicus*. J. Plant Physiol..

[96] Higuchi Y., Hisamatsu T. (2015). CsTFL1, a constitutive local repressor of flowering, modulates floral initiation by antagonising florigen complex activity in chrysanthemum. Plant Sci..

[97] Li C., Fu Q., Niu L., Luo L., Chen J., Xu Z.F. (2017). Three *TFL1* homologues regulate floral initiation in the biofuel plant *Jatropha curcas*. Sci. Rep..

[98] Oda A., Narumi T., Li T., Kando T., Higuchi Y., Sumitomo K., Fukai S., Hisamatsu T. (2012). *CsFTL3*, a chrysanthemum flowering locus t-like gene, is a key regulator of photoperiodic flowering in chrysanthemums. J. Exp. Bot..

[99] Komiya R., Yokoi S., Shimamoto K. (2009). A gene network for long-day flowering activates *RFT1* encoding a mobile flowering signal in rice. Development.

[100] Kato-Noguchi H., Yasuda Y., Sasaki R. (2010). Soluble sugar availability of aerobically germinated barley, oat and rice coleoptiles in anoxia. J. Plant Physiol..

[101] Roldán M., Gómez-Mena C., Ruiz-García L., Salinas J., Martínez-Zapater J.M. (1999). Sucrose availability on the aerial part of the plant promotes morphogenesis and flowering of *Arabidopsis* in the dark. Plant J..

[102] Ohto M.A., Onai K., Furukawa Y., Aoki E., Araki T., Nakamura K. (2001). Effects of sugar on vegetative development and floral transition in *Arabidopsis*. Plant Physiol..

[103] Yu W., Liu Y., Song L., Jacobs D.F., Du X., Ying Y., Shao Q., Wu J. (2017). Effect of differential light quality on morphology, photosynthesis, and antioxidant enzyme activity in *Camptotheca acuminata* seedlings. J. Plant Growth Regul..

[104] Aalifar M., Aliniaeifard S., Arab M., Zare Mehrjerdi M., Dianati Daylami S., Serek M., Woltering E., Li T. (2020). Blue light improves vase life of carnation cut flowers through its effect on the antioxidant defense system. Front. Plant Sci..

[105] Nishihara E., Kondo K., Parvez M.M., Takahashi K., Watanabe K., Tanaka K. (2003). Role of 5-aminolevulinic acid (ALA) on active oxygen-scavenging system in NaCl-treated spinach (*Spinacia oleracea*). J. Plant Physiol..

[106] Luthria D.L., Mukhopadhyay S., Krizek D.T. (2006). Content of total phenolics and phenolic acids in tomato (*Lycopersicon esculentum* Mill.) fruits as influenced by cultivar and solar UV radiation. J. Food Compos. Anal..

[107] Johkan M., Shoji K., Goto F., Hashida S.N., Yoshihara T. (2010). Blue light-emitting diode light irradiation of seedlings improves seedling quality and growth after transplanting in red leaf lettuce. HortScience.

[108] Vogt T. (2010). Phenylpropanoid biosynthesis. Mol. Plant.

[109] Esnouf A., Latrille É., Steyer J.-P., Helias A. (2018). Representativeness of environmental impact assessment methods regarding life cycle inventories. Sci. Total Environ..

[110] Ohl S., Hedrick S.A., Chory J., Lamb C.J. (1990). Functional properties of a phenylalanine ammonia-lyase promoter from *Arabidopsis*. Plant Cell.

[111] Helariutta Y., Elomaa P., Kotilainen M., Seppänen P., Teeri T.H. (1993). Cloning of cDNA coding for dihydroflavonol-4-reductase (DFR) and characterization of *dfr* expression in the corollas of *Gerbera hybrida* var. Regina (Compositae). Plant Mol. Biol..

[112] Pandey K.B., Rizvi S.I. (2009). Plant polyphenols as dietary antioxidants in human health and disease. Oxid. Med. Cell. Longev..

